# A non-interventional study of the genetic polymorphisms of NOD2 associated with increased mortality in non-alcoholic liver transplant patients

**DOI:** 10.1186/1471-230X-14-4

**Published:** 2014-01-06

**Authors:** Fuat Hakan Saner, Knut Nowak, Dieter Hoyer, Peter Rath, Ali Canbay, Andreas Paul, Michael Koldehoff, Ahmet Elmaağaclı

**Affiliations:** 1 Department of General- Visceral- and Transplant Surgery, University Duisburg-Essen, Hufelandstr 55, 45 122 Essen, Germany; 2 Department of Microbiology, University Duisburg-Essen, 45 122 Essen, Germany; 3 Department of Gastroenterology and Hepatology, University Duisburg-Essen, 45 122 Essen, Germany; 4 Department of Bone Marrow Transplantation, University Duisburg-Essen, 45 122 Essen, Germany

## Abstract

**Background:**

Infections after liver transplantation are the main cause of death in the first year. Recent reports indicate that NOD2 gene mutations increase the risk for inflammatory bowl disease and the severity of graft-versus-host disease in bone marrow transplant patients. Data on polymorphisms in liver transplant patients are sparse. We analyzed 13 single-nucleotide polymorphisms (SNPs) of 13 different gene variants including the SNPs of NOD2 genes from liver recipients. The aim of the study was to evaluate the impact of the SNPs on dialysis-dependent kidney failure, the incidence of infections and patient survival.

**Methods:**

During a period of 20-months, 231 patients were recruited in this non-interventional, prospective study. Thirteen different SNPs and their impact on the patients’ survival, infection rate, and use of dialysis were assessed.

**Results:**

NOD 2 wildtype genes were protective with respect to the survival of non-alcoholic, cirrhotic transplant patients (3 year survival: 66.8% wildtype vs. 42.6% gene mutation, p = 0.026). This effect was not observed in alcoholic transplant recipients.

The incidence of dialysis-dependent kidney failure and infection in the liver transplant patients was not influenced by NOD 2 gene polymorphisms. No effect was noted in the remaining 12 SNPs.

Patients with early allograft dysfunction experienced significantly more infections, required dialysis and had significantly worse survival.

In contrast, the donor-risk-index had no impact on the infection rate, use of dialysis or survival.

**Conclusion:**

NOD2 gene variants seem to play a key role in non-alcoholic, liver transplant recipients. However these data should be validated in a larger cohort.

## Background

Immunosuppression, as in liver transplant patients, carries a high risk of life threatening infections such as pneumonia or blood stream infections (BSI). Because calcineurin inhibitors are used as immunosuppressive agents to avoid organ rejection, infections have emerged in the last 20 years as the main cause of death in the first year after transplantation [1,2].

Several risk factors for infections after liver transplantation (LTX) have been identified, such as being male sex, having a prolonged cold ischemia time, acute liver failure, rejection treatment, relaparotomy due to bleeding or bile, and retransplantation within 30 days [3-5]. Recent reports indicate that single nucleotide polymorphisms (SNPs) are involved in a variety of diseases. Mutations of the toll-like receptor 4 (TLR-4) genes are associated with an increased incidence of inflammatory bowel disease due to bowl alterations such as changes in the bacterial load and translocation [6], whereas TLR-4 mutations in bone-marrow transplant patients are associated with an increased risk for graft-versus-host disease (GvHD) [7]. Nucleotide oligomerisation domain 2 (NOD2) polymorphisms, as a part of the innate immune system, have been shown to be protective against GvHD [7]. Several SNPs in specific genes of liver patients have been evaluated, with conflicting results. Mannose binding lectin (MBL) in the donor was found to carry a higher risk for postoperative life threatening infections [8,9]. Another clinical study indicates that an MBL gene polymorphism was associated with an increased risk for the development of hepato-cellular carcinoma in chronic hepatitis C patients [10]. The TLR 2 and TLR4-receptors are assumed to recognize hepatitis C virus. The association between TLR polymorphisms and outcome after transplantation due to chronic hepatitis C infection was evaluated in a clinical study [11]. The authors found that a homozygous TLR 2 polymorphism was associated with a higher incidence of poor graft function and higher mortality.

NOD2 gene polymorphism is associated with an increased incidence of liver and intestine failure in patients following combined liver and small bowl transplantation [12].

Recently, a group from Rotterdam [13] evaluated 50 polymorphisms in 181 liver transplant patients and their corresponding donors with respect to organ rejection and infections in the recipient. They found no correlation between the gene variants and bacterial and/or fungal infections.

The aim of our study was to evaluate the impact of 13 different SNPs, including in NOD2 and TLR 4, on transplant-associated complications such as infection, need for dialysis and the outcomes of the recipients.

## Methods

During the observation period of 2/2009 to 1/2012, a total of 231 patients were recruited in this prospective, non-interventional study. All patients received liver transplantations at the Medical Center University Essen, Department of General -, Visceral- and Transplant Surgery.

All patients were transplanted with whole liver from a brain-dead donor.

Exclusion criteria were recipient age ≤ 18 years and lack of a blood sample from the recipient.

This study was conducted in accordance with the amended Declaration of Helsinki. Local institutional review boards of the University Essen approved the protocol, and written informed consent was obtained from all patients.

### Surgical procedure and postoperative patient care

All transplants were accomplished with a liver from a deceased donor. Standardized surgical techniques were used for all recipients as was a strict anesthetic protocol.

A cell saver system was used for all patients who did not have hepatocellular carcinoma. Postoperatively, all patients were treated at a single intensive care unit (ICU) with standardized ICU treatment.

For immunosuppression, the patients received tacrolimus (0,1 mg/kg/d with a targeted plasma level of 5–8 ng/ml), mycophenolatmofetil (1000 mg twice a day) and intraoperative prednisolone (10 mg/kg, followed by 40 mg/day), which was gradually tapered within the 6 weeks after liver transplantation. Rejection episodes were treated with intravenous prednisolone (500 mg, 3 times/day).

Intraoperative antibiotic prophylaxis included intravenous ampicillin/sulbactam (3 grams), which was repeated if the operation time was longer than 3 hours. If there were no signs of infection, the antibiotic treatment was stopped after the surgical procedure. Selective digestive decontamination or fungal prophylaxis was not administered to the patients. A preventive treatment with valgancyclovir was performed in almost all patients, but not when both the donor and recipient were CMV IgG negative.

### Definitions

Infections (pneumonia and blood stream infections [BSI]) were diagnosed based on the criteria proposed by the Centers for Disease Control [14], as already described in one of our previously published studies [2]. Briefly, the diagnosis of pneumonia was based on the presence of pulmonary infiltrates together with clinical symptoms suggestive of a lower respiratory tract infection, the identification of a relevant etiologic microbial agent, and the absence of an alternative diagnosis during follow-up. Detection of *Aspergillus spp.* in the broncho-alveolar lavage was regarded as relevant and was adequately treated.

The diagnosis of BSI was defined as the isolation of gram-positive cocci, gram-negative rods, or any type of fungi in the blood culture.

Sepsis and pneumonia were summarized as clinical significant infections (CSI).

Mortality was defined as death from any cause during the hospital stay or within 1 year after discharge.

Early allograft dysfunction (EAD) was based on the EAD assessment published in 2010 [15]. Briefly, EAD was defined as: Bilirubin ≥10 mg/dL on postoperative day 7 and/or INR ≥1.6 on postoperative day 7 and/or AST or ALT >2000 IU/L within the first 7 days.

The donor-risk index (DRI) was assessed as previously described by Feng et al. [16].

### Polymorphism genotyping

DNA was prepared from the PBMCs obtained from the recipient after transplantation using the Magna Pure device from Roche diagnostics (Mannheim Germany). Genotyping for the NOD2/CARD15 gene (SNP8, R702W, rs2066844, SNP12, G908R, rs2066845 and SNP 13, 1007 fs, rs2066847) was performed with a Taqman protocol, as published previously [7,17]. Genotyping for TLR 4 (D299G; rs4986790) and TLR 4 (T399I; rs4987233) was performed and then analyzed using hybridization probes and primers, as previously published [18]. The call rate in a Taqman Genotyper Software study for genotyping SNP 8, SNP 12, and SNP 13 was 90% for each. All tested polymorphisms are listed in Table 1.

**Table 1:** Polymorphisms (SNPs) tested in all liver transplant patients

	
IL 23R	rs11209026
IL 18 RAP	rs917997
TLR1	rs5743611
TLR4	rs4986790
TLR9	rs187084
CARD 9	rs4077515
NQO	rs1800566
CYP1B1 codon 432	rs1056836
MTHFR1298	rs1801131
MTHFR 677	rs180133
NOD2 G908R	rs2066847
NOD2 L1007F insC	rs20666847
NOD2 R702W	rs2066844

The fluorescence-labeled sensor and anchor hybridization probes for the Lightcycler protocol and Taqman probes were purchased from TIB MOLBIOL (Berlin, Germany). Sensor probes cover for the SNPs and exhibit a different temperature depending on binding to the wild type or variant allele. Anchor probes were designed to display a significantly higher melting temperature compared with the corresponding sensor probes. PCR and subsequent melting curve analysis were performed using software for the Lightcycler device. Control samples were included in each run [17].

We included the allele and genotype of the examined SNPs with prefabricated LightSNiP arrays (TIB MOLBIOL GmBH, Berlin, Germany). The 96-well array was company technically with the plate configuration like NOD2, TLR1,-4,-9 and other SNPs compiled. The candidate genotypes were selected mainly based on the literatures that were available on 2009/2010 when the study was initially designed. The allele frequencies are compared with reference population (http://www.ncbi.nlm.nih.gov and http://www.hapmap.org) for Caucasian. Genotype probabilities can then be calculated using Hardy-Weinberg equilibrium (HWE) assumption or other assumptions that relate allele frequencies to the allele frequencies.

### Statistical analysis

Continuous data are given as the mean with the standard deviation (SD). Dichotomous or categorical variables are given as numbers with percentage. Differences in continuous variables were calculated using the student’s t-test and differences in categorical variables were compared using the Pearson’s Chi squared test. Kaplan-Meier curves were constructed. A p-value < 0.05 was considered statistically significant. Statistical analysis was performed using SPSS statistical software version 20.0 for Mac (SPSS Inc, Chicago III, USA).

## Results

### Clinical characteristics and post-transplant clinical significant infections

From 2/2009 – 01/2012, a total of 231 post-liver transplant patients (67.5% m, 32.5% f) were recruited into the study.

The diagnosis leading to liver transplantation were given in Table 2.

**Table 2:** Diagnosis leading to transplantation

**Diagnosis**	**Number of patients**
**Alcoholic related cirrhosis**	60
**Hepatitis C**	63
**Hepatitis B**	22
**Non-alcoholic-steatohepatitis (NASH)**	15
**Acute liver failure**	7
**Primary sclerosing cholangitis**	11
**Cryptogenic**	12
**Polycystic Liver disease**	6
**Haemochromatosis**	3
**Wilson disease**	5
**Autoimmunhepatitis**	6
**Hepato-cellular cirrhosis**	3
**Secondary sclerosing hepatitis**	7
**Primary biliary cirrhosis**	3
**Miscellaneous**	8
**Total**	231

Miscellaneous: Budd-Chiari Syndome (1); Amyloidosis (2), Cholangiocellular carcinoma (1); Embryonal sarcoma (1); Neuroendocrine tumor (1), secondary biliary cirrhosis (1); alpha 1 antitrypsine deficiency (1).

The CSI rate was 26.4%, which significantly affected the ventilation time; stay in the ICU; the incidence of dialysis use; and survival rate. The median ventilation time for patients with CSI was 198 h (8–3035 h) compared to 20 h (0–617) in patients without CSI (p < 0.0001). CSI resulted in a median ICU stay of 18 days (2–130), which was significantly longer than the 4 days (1–27) in patients without CSI (p < 0.0001). The incidence of dialysis-dependent kidney failure increased from 26.4% in patients without infections to 60.6% in patients with CSI (p < 0.0001). The rate of dialysis in the whole cohort was 32.3%. Table 3 details the clinical and demographic characteristics of both cohorts, patients with and without CSIs.

**Table 3:** Clinical variables and characteristics for both cohorts (with and without CSI)

	**Patients without CSI**	**Patients with CSI**	**P value**
Age	52 ± 9	53 ± 10	0.8
MELD	21 ± 9	25 ± 11	0.006
Ventilation time (in hours)	20 (0–617)	198 (8–3035)	< 0.001
ICU stay (days)	4 (1–27)	18 (2–130)	< 0.001
Hospital stay (days)	21 (2–136)	42 (3–244)	< 0.001
Incidence of EAD	18.2%	38.7%	< 0.001
DRI	1.8 ± 0.38	1.7 ± 0.36	0.16
WIT (min)	34 ± 12	33 ± 7	0.7
CIT (min)	427 ± 136	461 ± 154	0.1
Incidence of Dialysis	26.4%	60.6%	< 0.001

CSI: clinical significant infections.

ICU: intensive care unit.

EAD: early allograft dysfunction.

DRI: donor-risk-index.

WIT: warm ischemia time.

CIT: cold ischemia time.

The 1-year survival rate decreased in patients with CSIs to 42% compared with 83.7% in patients without infection (p = 0.0001). The 1-year-survival for all patients was 72.6%.

The donor-risk-index (DRI) did not affect either the infection or survival rates. Patients with CSI had a significantly higher lab MELD than patients without CSI (25 ±10 vs. 21 ± 9, p = 0.006).

EAD after transplantation affected both the CSI and survival rates. The CSI rate in patients with EAD was 38.7% compared to 18.2% (p = 0.001) in patients without EAD. Survival decreased from 78.1% to 64.7% (p = 0.023) in patients with severe infection.

### Genetic polymorphisms in the non-interventional study group and their impact on infection and survival

The allele and genotype distribution of the SNPs in the different groups (alcoholic and non-alcoholic) were given in the Tables 4 and 5.

**Table 4:** **Characteristics of genes and polymorphisms** → **patients (Pts) with alcoholic liver failure**

**Gene**	**Allele**	**rs number**	**Wild type genotype**	**Heterozygote genotype**	**Homozygote mutation**	**Pts**
			**n (%)**	**n (%)**	**n (%)**	
IL23R	G/A	11209026	44 (85)	8 (15)		52
IL18 RAP	A/G	917997	25 (48)	23 (44)	4 (8)	52
CARD9	G/A	4077515	21 (40)	23 (44)	8 (15)	52
NOD2	C/T	2066844	40 (77)	12 (23)		52
NOD2	G/C	2066845	48 (80)	10 (17)	2(3)	60
NOD2	-/C	2066847	47 (90)	4 (8)	1 (2)	52
TLR1	G/C	5743611	32 (62)	14 (27)	6 (12)	52
TLR4 299	G/A	4986790	50 (96)	1 (2)	1 (2)	52
TLR4 399	G/A	4987233	48 (92)	3 (6)	1 (2)	52
TLR9 1237	C/T	5743836	16 (31)	27 (53)	8 (16)	51
TLR9 1486	T/C	187084	31 (61)	19 (37)	1 (2)	51
NQO1 609	C/T	1800566	41 (79)	9 (17)	2 (4)	52
CYP1B1 432	G/C	1056836	16 (31)	20 (39)	15 (30)	51
MTHFR 677	T/C	1801133	23 (45)	22 (43)	6 (12)	51
MTHFR 1298	C/A	1801131	18 (41)	23 (52)	3 (7)	44

**Table 5:** Characteristics of genes and polymorphisms → patients (Pts) with non-alcoholic liver failure

**Gene**	**Allele**	**rs number**	**Wild type genotype**	**Heterozygote genotype**	**Homozygote mutation**	**Pts**
			**n (%)**	**n (%)**	**n (%)**	
IL23R	G/A	11209026	138 (93)	11 (7)		149
IL18 RAP	A/G	917997	75 (51)	61 (41)	12 (8)	148
Card9	G/A	4077515	59 (40)	67 (45)	22 (15)	148
NOD2	C/T	2066844	128 (86)		21 (14)	149
NOD2	G/C	2066845	138 (81)	22 (13)	11 (6)	171
NOD2	-/C	2066847	145 (97)	3 (2)	1 (1)	149
TLR1	G/C	5743611	82 (55)	56 (38)	11 (7)	149
TLR4 299	G/A	4986790	128 (86)	19 (13)	2 (1)	149
TLR4 399	G/A	4987233	130 (87)	18 (12)	1 (1)	149
TLR9 1237	C/T	5743836	50 (34)	68 (46)	29 (20)	147
TLR9 1486	T/C	187084	90 (61)	47 (32)	10 (7)	147
NQO1 609	C/T	1800566	92 (62)	47 (31)	10 (7)	149
CYP1B1 432	G/C	1056836	59 (40)	63 (42)	26 (18)	148
MTHFR 677	T/C	1801133	58 (39)	66 (45)	23 (16)	147
MTHFR 1298	C/A	1801131	67 (53)	42 (33)	18 (14)	127

**Figure 1:**
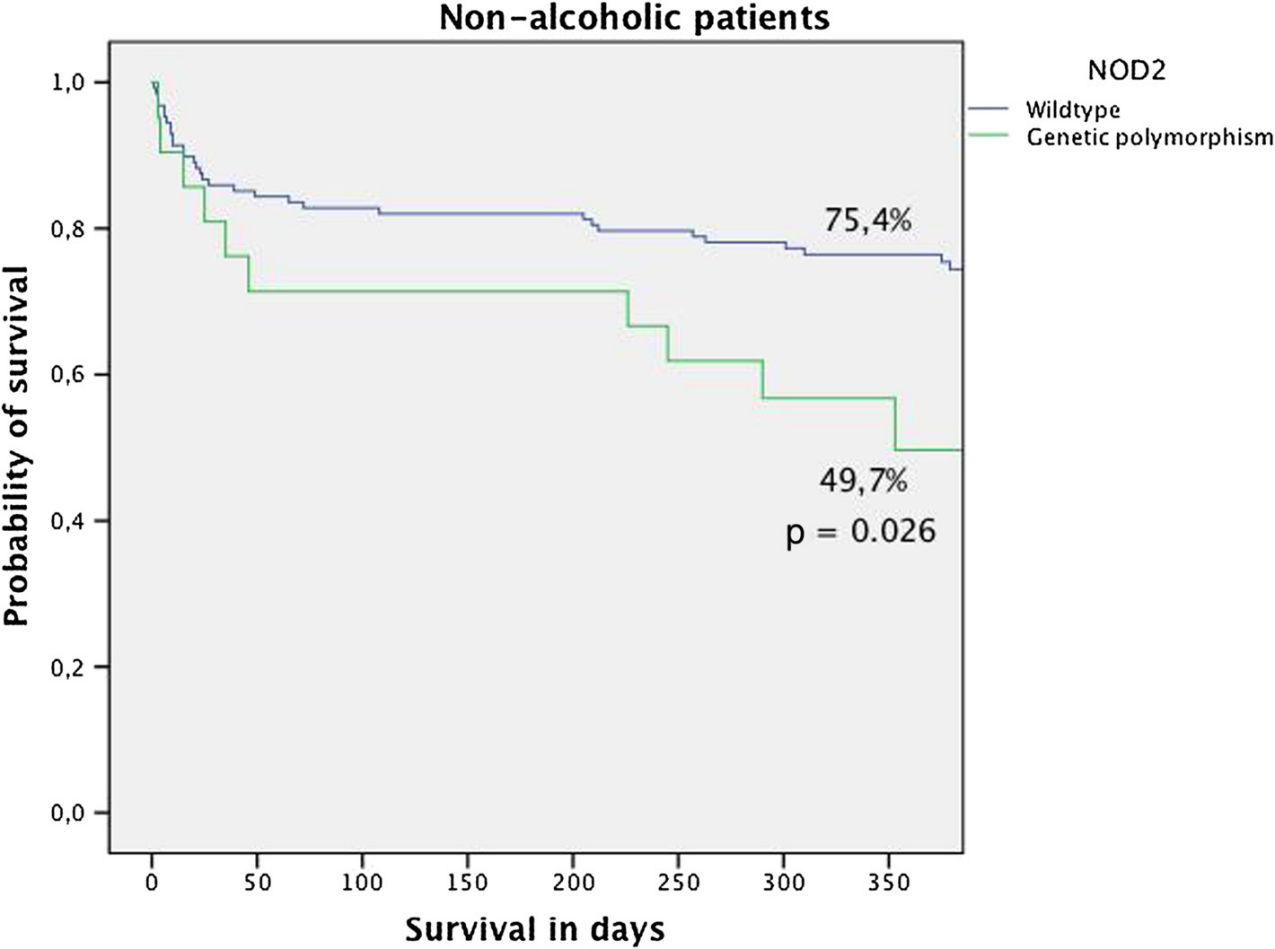
**Presents the 1-year survival of patients who received liver transplants for non-alcoholic liver disease.** The wild type NOD2 is protective with respect to the survival rate in this cohort. The 1–year survival rate for patients with wild type NOD2 was 75.4% compared to 49.2% in patients with the gene variant.

Non-alcoholic patients received a survival benefit from NOD 2 with wild type variants. Non-alcoholic patients with wild type NOD2 genes had a one-year survival rate of 75.4%, whereas the patients with polymorphisms had a survival rate of 49.7% (p = 0.026) (Figure 1). The difference between wild type NOD2 and polymorphism variants became significant at 6 months after transplantation. This was not the case in alcoholic patients who received liver transplants. The observed 1-year survival in patients with alcoholic disease and a genetic polymorphism for NOD 2 was 83%, which was higher than that in the NOD2 wild type group (64%), but this did not reach statistical significance. The overall survival for the whole population (alcoholic and non-alcoholic patients) was 72.7% for the wild type NOD2 vs. 62.5% for the genetic polymorphism (p = 0.45). Table 6 presents the clinical characteristics of the non-alcoholic and alcoholic patients. Patients with non-alcoholic disease were significantly older and required significantly more dialysis treatment.

**Table 6:** Clinical variables in alcoholic and non-alcoholic patients

	**Non-alcoholic disease**	**Alcoholic disease**	**P value**
age	52 ± 10	55 ± 8	0.017
CIT (minutes)	447 ± 145	406 ± 124	0.048
WIT (minutes)	33 ± 9	34 ± 15	0.7
MELD	22 ± 9	21 ± 8	0.38
Incidence of EAD	44.4%	28.8%	0.024
Incidence of CSI	25.7%	28.3%	0.4
Incidence of Dialysis	35.3%	23.7%	0.07
DRI	1.8 ±0.4	1.8 ± 0.3	0.6
Ventilation time (hours)	35 (0–1681)	26 (0–3035)	0.2
ICU stay (days)	5.5 (1–71)	5 (1–130)	0.4
Hospital stay	23.5 (2–244)	28 (3–169)	0.3

ICU: Intensive Care Unit.

EAD: early allograft dysfunction.

DRI: donor-risk-index.

WIT: warm ischemia time.

CIT: cold ischemia time.

CSI: clinical significant infections.

NOD-2 polymorphism did not affect the incidence of infection (28.6% vs. 30.3%) or incidence of dialysis (33.2% vs. 30.3%). However, NOD 2 polymorphism seems to be protective against EAD. Patients with NOD-2 wild type developed EAD in 44.3% of the cases, whereas patients with the gene variant had a moderate EAD incidence of 24.2% (p = 0.024).

None of the other SNP gene variants had significant results in the evaluated parameters.

## Discussion

Our study indicates that wild type NOD2 confers a survival benefit to non-alcoholic patients who receive a liver transplant. We recorded a 36% higher survival in patients with the wild type gene variants compared to the NOD 2 mutation gene variants in this cohort. However, these results were not reproducible for patients with alcoholic disease. In contrast, in patients with alcoholic disease, the gene variant of NOD2 seems to increase survival, though this finding was not statistically significant. Patients with alcoholic disease were significantly older according to statistical analysis, but the difference of 3 years (55 vs. 52 years) seems clinically negligible.

EAD is a serious complication after liver transplantation, which is associated with a higher retransplantation rate and higher mortality [19-21]. EAD was almost 2 times more common in patients with non-alcoholic disease compared to alcoholic patients who received a liver transplant. However, the protective effect of the NOD2 gene with wild type variants overruled the EAD effect.

Recent reports have provided evidence that NOD2 gene variants influence the severity of graft-versus-host-disease (GVHD) and the transplant survival in bone marrow transplant (BMT) patients.

The Elmaagacli group demonstrated a significantly higher incidence of GVHD in patients with gene variants of NOD2 following BMT compared with patients who had wild type NOD2 [7]. These data are in agreement with a study by Holler et al. [22] in which there was an increased risk of GVHD in patients with an NOD2 gene variant. The authors also reported an increased risk of mortality with the gene variant, which is in contrast with the findings of the Elmaagacli group [7].

The Pittsburgh group evaluated the NOD 2 gene variant in simultaneous liver and small bowel transplanted pediatric patients [12]. They reported that NOD2 gene variants were associated with increased liver and intestinal failure in patients with short-gut syndrome.

Although NOD2, as a part of the innate immune system, plays a key role in preventing bacterial infections, we did not find a difference among patients with and without CSI. Moreover, NOD2 mutations were not associated with a decreased incidence of dialysis use; wild type NOD2 in non-alcoholic patients may have an independent protective effect for survival.

More than a quarter of our patients experienced CSI, which was associated with significantly higher rates of dialysis use, longer ventilation time, length of ICU – and hospital stay, and higher mortality. In an earlier study conducted by our department, the rate of CSI (pneumonia and sepsis) was 32.3% (24% sepsis rate and 8.3% pneumonia rate) [2]. The reason for the higher CSI rate may be related to a different patient population. While only patients with a postmortem organ were recruited in the current study, 24% of patients in the previous study were transplanted with a graft from a living donor. Biliary complications, including bile leaks, which are associated with sepsis, range from 0.3 - 22% in whole organ transplantation [23,24], which is significantly lower than with split liver transplantation with a living donor graft. Some groups reported biliary complications in split liver transplantation of up to 60% [25], which may contribute to a lower sepsis rate in the current study despite a higher MELD score compared with our own data in the last decade.

A recently published study [26] evaluated the risk factors for infections in 367 patients. The authors reported a CSI (pneumonia and blood stream infection) rate of 28.8%, which is similar to the incidence in our study. In line with this study, Sganga et al. [27] identified a blood stream infection rate of 28%. Unfortunately, neither study reported the survival rate in patients with infection. Our study indicated a nearly 50% decrease in the one-year-survival for patients who develop a CSI, which is significantly higher than in other groups. Sun et al. [28] evaluated the risk factors for infections in the MELD era. The group stated that the recipient age (OR =1.09) and the MELD score affect infection. They reported an overall (patients with and without infections) one-year survival rate of 76.7% in the Pre-MELD era and 82.5% in the MELD era. In our study, the one-year survival rate was 72.3% for all patients. The MELD scores in both studies were comparable, but there is a difference of 10% in the one-year survival of all patients, which may be related to the quality of the donor graft. While in the UNOS region, only 32% of the transplanted grafts have a DRI of > 1.5; this rate accounts for 65% in the EUROTRANSPLANT region. Moreover, only 6% of the transplanted organs in the UNOS region have a DRI > 2, but 23% in the EUROTRANSPLANT area have a DRI > 2, which is associated with a higher graft failure and lower patient survival [29]. This may in part explain the higher mortality.

There are several studies reporting a worse outcome of liver transplantation in patients with kidney dysfunction and dialysis [30-32]. The registry data of liver transplant patients with postoperative kidney dysfunction demonstrate a significantly worse 2-year survival compared with patients with adequate or mild kidney dysfunction (55% vs. 76%, p < 0.05) [33]. These data are in line with a retrospective study that evaluated the risk factors in terms of the survival rate after liver transplantation [34]. Patients with dialysis-dependent kidney failure had a one-year survival of less than 50%.

These data are comparable with our study. Sixty-six percent of patients with CSI had dialysis-dependent kidney failure, which was associated with a one-year survival 64.7%. Sun et al. reported of a dialysis-dependent kidney failure in less than 10% of their transplanted patients. In contrast to that study, 32.3% of our patients required dialysis treatment due to kidney failure, which affects their outcomes. In this context, it is indispensible to highlight the significantly higher dialysis rate in patients with non-alcoholic disease than in alcoholic patients in our cohort. Despite this fact, wild type NOD2 was protective in this patient cohort with respect to their long-term survival.

Our study has some limitations. First, it was a single-center study. Because we conducted an observational study, there is no real control group. We did not stratify or evaluate the patients’ outcomes with respect to the transplant surgeon or anesthesia team. However because 2007 we have had a constant dedicated surgery and anesthesia team who take care for these patients.

## Conclusion

In conclusion, our data indicates wild type NOD2 offers a survival benefit in liver transplant patients with non-alcoholic disease. Patients with the NOD2 gene variant should have closer follow-up as part of the standard of care. The other SNPs that we tested did not significantly influence the CSI and survival rates in this cohort. Transplant-related factors may be more dominant risk factors for these patients than the genetic polymorphisms.

### Key messages

1. NOD2 gene polymorphism is associated with higher mortality in patients non-alcoholic disease.

2. DRI has no impact on infection and survivial.

3. EAD is associated with significantly more CSI.

4. Patients with CSI had a significant worse 1-year survival, more common dialysis treatment, longer ventilation time, and longer ICU stay compared to patients without infection.

## Abbreviations

NOD2: Nucleotide-binding oligomerization domain-containing protein 2; SNP: Single nucleotide polymorphism; LTX: Liver transplantation; TLR-4: Toll-like receptor 4; GvHD: Graft-versus-host-disease; ICU: Intensive care unit; IgG: Immunglobulin G; DRI: Donor-risk-index; EAD: Early-allograft-dysfunction; AST: Asparagin transaminase; ALT: Alanine transaminase; ET: Eurotransplant; UNOS: United network for organ sharing; CSI: Clinical signifcant infection; WIT: Warm ischemia time; CIT: Cold ischemia time.

## Competing interests

The authors declare that they have no competing interests.

## Authors’ contributions

FS has made substantial contributions to conception and design, acquisition of data and analysis. KN has made substantial contributions to acquisition of data and analysis. DH has made substantial contributions to acquisition of data and analysis. PR had important intellectual contribution for the study design. AP has provided final approval of the version to be published. AC has made substantial contributions to conception, design analysis and interpretation of data. MK has made substantial contributions to conception, design analysis and interpretation of data. All authors read and approved the final manuscript.

## Pre-publication history

The pre-publication history for this paper can be accessed here:

http://www.biomedcentral.com/1471-230X/14/4/prepub
